# Electrophile Signaling and Emerging Immuno- and Neuro-modulatory Electrophilic Pharmaceuticals

**DOI:** 10.3389/fnagi.2020.00001

**Published:** 2020-02-07

**Authors:** Jesse R. Poganik, Yimon Aye

**Affiliations:** Swiss Federal Institute of Technology Lausanne (EPFL), Lausanne, Switzerland

**Keywords:** lipid-derived electrophiles, dimethyl fumarate, central nervous system, 4-hydroxynonenal, multiple sclerosis

## Abstract

With a lipid-rich environment and elevated oxygen consumption, the central nervous system (CNS) is subject to intricate regulation by lipid-derived electrophiles (LDEs). Investigations into oxidative damage and chronic LDE generation in neural disorders have spurred the development of tools that can detect and catalog the gamut of LDE-adducted proteins. Despite these advances, deconstructing the precise consequences of individual protein-specific LDE modifications remained largely impossible until recently. In this perspective, we first overview emerging toolsets that can decode electrophile-signaling events in a protein/context-specific manner, and how the accumulating mechanistic insights brought about by these tools have begun to offer new means to modulate pathways relevant to multiple sclerosis (MS). By surveying the latest data surrounding the blockbuster MS drug dimethyl fumarate that functions through LDE-signaling-like mechanisms, we further provide a vision for how chemical biology tools probing electrophile signaling may be leveraged toward novel interventions in CNS disease.

## Introduction: Electrophile Signaling in the CNS

Both endogenous reactive electrophilic metabolites [e.g., lipid-derived electrophiles (LDEs)] and exogenous electrophilic agents are particularly relevant to the central nervous system (CNS). The abundance of polyunsaturated fatty acids (PUFAs) in neuronal membranes, together with heightened reactive oxygen species (ROS) generation caused by high oxygen consumption, drives LDE production (Chen et al., 2008). Further elevated buildup is implicated in neurodegenerative diseases (Zarkovic, 2003). Although not the focus of this perspective, ROS levels are positively correlated with LDE levels, as ROS can oxidize PUFAs, generating LDEs (Schopfer et al., 2011). Abundance of redox-active metals in neuronal tissues further assists LDE generation. By contrast, the key enzymes involved in LDE detoxification (e.g., glutathione S-transferases) are expressed at low levels in mammalian brain tissue (Zheng et al., 2014). Collectively, these factors render the CNS rife with LDEs. Indeed, 4-hydroxynonenal (HNE, a prototypical LDE) accumulates to a greater extent (approximately 2-fold) in the brain than in other tissues in mice (Sultana et al., 2013). Other endogenous electrophilic metabolites, such as fumarate (a product of the TCA cycle; Raimundo et al., 2011; Kulkarni et al., 2019) and itaconate (produced by Irg1 in macrophages from *cis*-aconitate generated by the TCA cycle; Michelucci et al., 2013; Qin et al., 2019) are also increasingly implicated as CNS signaling mediators (Daniels et al., 2019). Great effort has thus been put forth to develop tools with which to study LDEs and their protein targets.

## High-Throughput Chemoproteomics Methods to Profile Potential LDE Sensors

**Figure 1:**
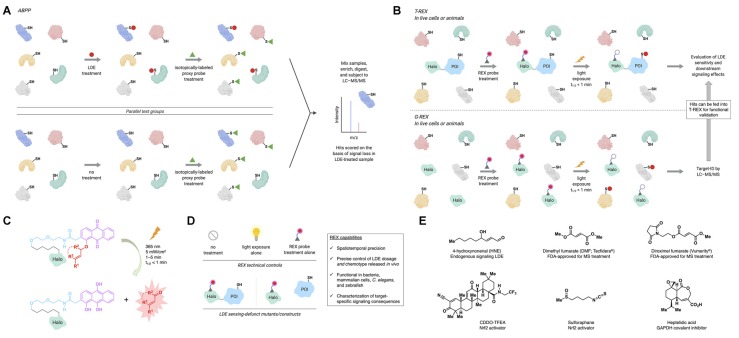
Activity-based protein profiling (ABPP) and REX technologies profile lipid-derived electrophile (LDE) sensors and/or interrogate target-specific LDE signaling. **(A)** In ABPP, parallel test groups (typically lysates/homogenates) are first treated with the LDE of interest, or not treated (typically, DMSO control). Subsequently, both groups are treated with a broadly reactive proxy electrophile probe, which is isotopically labeled. The samples are then mixed, enriched, digested, and subjected to liquid chromatography–tandem mass spectrometry (LC–MS/MS), where loss of proxy probe labeling allows quantitative ranking of LDE modification events. **(B)** Top: in targetable reactive electrophiles and oxidants (T-REX), a Halo-protein of interest (POI) fusion is expressed in live cells, worms, or zebrafish. The system is then treated with a photocaged precursor to an LDE of interest (REX probe; see panel **C**). After removal of excess REX probe, the system is exposed to UV light (365 nm, 5 mW/cm^2^, 1–5 min) to liberate, in the vicinity of the POI, the LDE (in an amount maximally stoichiometric to the *in vivo* concentration of Halo-POI). Provided the POI is a kinetically privileged sensor (KPS) of the LDE, it will react before the LDE diffuses away. LDE-sensing ability and downstream signaling effects can then be assayed by a number of downstream procedures (Poganik et al., 2019a). Bottom: genome-wide profiling ofreactive-electrophile and -oxidant sensors (G-REX) is similar to T-REX except that G-REX involves expression of HaloTag with no POI fusion. The liberated LDE (with maximum dosage equivalent to *in vivo* HaloTag concentration) is captured by endogenous KPSs, which are profiled by standard quantitative proteomics (e.g., SILAC, TMT) following enrichment and digest (Poganik et al., 2019a). Hits identified by G-REX can then be fed into the T-REX workflow to validate their LDE-sensing ability and investigate target-specific consequences of LDE modification. **(C)** REX probes are modular, bio-inert, bind selectively and irreversibly to HaloTag *in vivo*, and allow rapid release of LDEs on demand. **(D)** Technical controls in applying REX techniques include no treatment, light exposure alone, and REX probe treatment alone. Functional controls in applying T-REX include LDE-sensing-defunct mutant POIs (by mutation of the LDE-sensing cysteine) and split constructs where Halo and POI are expressed separately (conditions under which the POI cannot be LDE-modified upon T-REX). Inset: capabilities of REX inaccessible by other tools. **(E)** Structures of select endogenous signaling LDEs and electrophilic drugs and inhibitors discussed in the text.

As methods to examine LDE modifications have been reviewed extensively (Parvez et al., 2018), we focus only on select target identification and signaling-validation/interrogation tools. From the initial identification (in 1980) of HNE as a product of microsomal lipid peroxidation (Benedetti et al., 1980) to mid-2013, all strategies to probe LDE signaling relied on global administration of LDEs, which modifies many targets simultaneously. Among the bolus regimens, activity-based protein profiling (ABPP), pioneered in the 1990s, remains a go-to platform for target ID (Liu et al., 1999). The ABPP workflow to profile potential LDE-sensitive proteins (Wang et al., 2014) consists of parallel test groups: globally LDE-treated (typically in lysates/homogenates) samples; and non-treated controls. Both groups are subsequently globally treated with a putatively more promiscuous, isotopically labeled proxy electrophile probe, featuring an enrichment handle (e.g., biotin). Following enrichment, peptide digest, and liquid chromatography–tandem mass spectrometry (LC–MS/MS), hits are scored based on the ratio of their detection in the two groups (Figure 1A). ABPP is the first high-throughput platform capable of ranking hits quantitatively, while enabling the identification of LDE modification sites. However, because ABPP relies on an indirect/loss-of-signal readout, different target spectra are obtained depending on the identity of proxy probe deployed, even for the same LDE assessed (Wong and Liebler, 2008; Abegg et al., 2015). Setup in lysates lacks biological context, and analyses are inherently biased toward the detection of high-occupancy LDE modifications. Recent advances in proteomic profiling techniques have attempted to obviate some of the limitations of traditional ABPP through, for instance, elimination of the need for proxy probe treatment (Chen et al., 2017, 2018).

## Rex Technologies Profile LDE-Responsive Targets at Low-Occupancy and Probe On-Target LDE Signaling

To address the longstanding problem underlying the lack of protein-specific tractability in LDE signaling, targetable reactive electrophiles and oxidants (T-REX) was introduced in mid-2013 (Fang et al., 2013; Figure 1B). T-REX employs a HaloTag-fused protein of interest (POI) and a photocaged precursor to a specific LDE (REX probe hereafter). These biologically inert REX probes bind specifically and irreversibly to HaloTag (Figure 1C). Upon light exposure, the LDE is liberated in the vicinity of the Halo-POI. If the POI is a sensor of the LDE, it is captured before diffusion away from the POI. T-REX thus provides a previously inaccessible opportunity to trace LDE information flow at the POI-specific level. It further informs quantitatively on ligand occupancy and modification site ID (Parvez et al., 2016). With a clear link between occupancy and on-target signaling, T-REX has shown that a subset of LDE sensor proteins *does not require high RES occupancy to evoke a dominant signaling response*: such proteins are termed kinetically privileged sensors (KPSs; Liu et al., 2019). LDE occupancy ranges from ~10% to 30% for several KPSs studied, explaining why they are not scored as hits in ABPP. To enable high-throughput searches for KPSs, G-REX (global reactive electrophiles and oxidants) was developed (Zhao et al., 2018). With omission of POI fusion, LDE released from HaloTag is captured by KPSs. Standard enrichment and proteomics procedures enable rapid target ID. Critically, hits can be faithfully validated by T-REX for LDE sensitivity and signaling propensity (Figure 1B). As the amount of LDE delivered *in vivo* is tunable by adjusting the expression level of the Halo (POI), intracellular LDE dosage is controlled. Control of HaloTag-expression locale and light-exposure time offers spatiotemporal resolution. REX approaches do rely on HaloTag overexpression (with or without POI fusion) and UV-light exposure (1–5 min at 5 mW/cm^2^); however, potential artifacts are controlled for by a suite of technical controls and RES-sensing-defunct-but-otherwise-functional mutant POIs/constructs (Figure 1D).

## LDE Regulation of Keap1/Nrf2/Antioxidant Response (AR) Signaling in Multiple Sclerosis (MS)

Emerging classes of broad-specificity covalent drugs featuring electrophilic motifs similar to those in LDEs have recently received FDA approval or entered clinical/preclinical trials for CNS-related diseases, e.g., MS (Figure 1E). MS is an incurable autoimmune disease characterized by chronic inflammation of the CNS. Plaque-like inflammatory lesions lead to damage of myelin sheaths, the protective, insulating coating of neurons (Reich et al., 2018). This damage ultimately produces the neurologic disabilities MS patients experience. MS lesions contain macrophages, T cells, antibodies, and complement (Lucchinetti et al., 2000). Interestingly, mutations in immune genes comprise the largest group of genetic risk factors identified for MS (International Multiple Sclerosis Genetics Consortium et al., 2011). Thus, the immune system, which is itself heavily modulated by LDE signaling, is critically important in MS development/progression. Based on our latest understanding of electrophile signaling in the CNS and immune system, we here discuss how LDE regulation interplays with Keap1/Nrf2/AR signaling, a major stress defense pathway implicated in MS.

The transcription factor Nrf2 drives the expression of a suite of antioxidant/detoxifying genes to mount a cytoprotective response, the AR (Hayes and Dinkova-Kostova, 2014). Nrf2-protein is activated in response to LDE modification of Keap1, the negative regulator and cytosolic anchor of Nrf2. Nuanced regulatory mechanisms of Nrf2–mRNA under stress are also increasingly appreciated (Poganik et al., 2019b) but poorly understood. Nrf2 plays key roles in CNS and autoimmune disease, particularly MS, through suppression of inflammation (Cuadrado et al., 2019). In MS patients, Nrf2 activation is a good predictor of therapeutic response to some MS drugs (Hammer et al., 2018). Evidence for a protective effect of Nrf2 in MS has been demonstrated in experimental autoimmune encephalopathy (EAE) mice (a widely used MS model), where activation of Nrf2 by electrophiles (e.g., sulforaphane; Figure 1E) significantly attenuates disease development/progression (Johnson et al., 2010; Li et al., 2013; Kobayashi et al., 2016). The benefit of treating a rat EAE model with dihydro-CDDO-trifluoroethyl-amide (CDDO-TFEA, a bardoxolone-methyl derivative and Nrf2 activator; Figure 1E) appears to extend to remyelination of damaged neurons (Pareek et al., 2011).

Despite these significant findings, studies involving global administration of reactive electrophiles fail to render unambiguous links between target engagement and signaling/therapeutic output. Our lab thus began to address some of these key questions. Applying T-REX against various controls documents that substoichiometric HNEylation of Keap1 alone is sufficient to trigger AR (Parvez et al., 2015, 2016). This finding opens the possibility of designing Keap1-selective electrophilic agents to upregulate Nrf2/AR. Because REX probe design is modular (Figure 1C), mechanisms-of-action (MOAs) and structure-activity relationships (SARs) of novel candidates can be studied using T-REX. Another conflicting aspect surrounding the Nrf2/AR pathway in CNS disease lies in the benefit of Nrf2 activation vs. inhibition. Many Nrf2 inhibitors and inducers have reached late-stage clinical trials, although context-specific aspects (cell type, subcellular locale, disease stage/subtype, etc.) of Nrf2 regulation remain unsolved (Cuadrado et al., 2019). For instance, low Nrf2 expression levels in neurons imply that neuroprotective effects of Nrf2 activation in MS lesions arise from another cell type (van Horssen et al., 2010; Licht-Mayer et al., 2015). T-REX-assisted Keap1-specific modification executed in particular cell types and parallel assessment of inter- vs. intracellular Nrf2/AR pathway communications are a promising way of shedding light on these unresolved questions. Because T-REX is a transient electrophile-delivery procedure, it is particularly suited to tackling these questions in sensitive cells, where classical genetic approaches involving persistent changes to gene expression may be detrimental. These examples underscore the need for understanding context-specific LDE regulation in disease. As we relate below, the recent emergence of broadly reactive electrophilic MS drugs further highlights the paramount importance of understanding electrophile signaling.

## Dimethyl Fumarate (DMF) in the Treatment of MS

**Figure 2:**
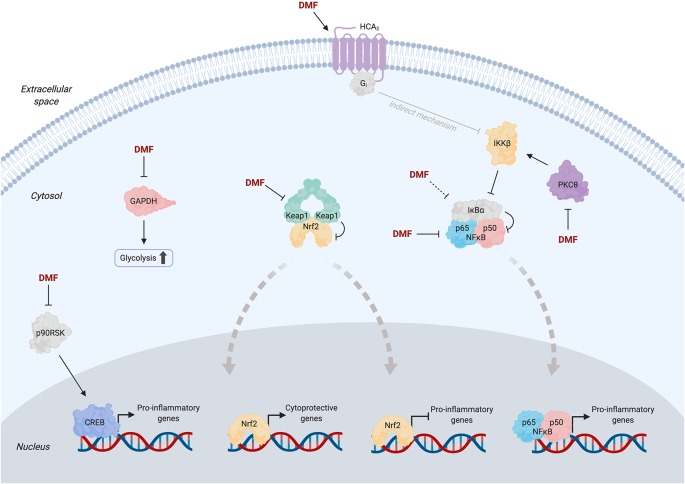
Simplified pathway diagram showing selected proposed targets of dimethyl fumarate (DMF) and potential associated mechanisms at the molecular level. Solid arrows from DMF indicate literature evidence for direct binding to the target; dashed arrows indicate purported targets for which evidence of direct binding has not yet been provided (see text for detailed discussions). Thick dashed gray arrows indicate nuclear translocation events. Apart from Keap1, oligomeric states of proteins are depicted as monomeric for simplicity. Transcriptional co-regulators of Nrf2, NFκB, and CREB are omitted for clarity. Note that the mechanism of inhibition of NFκB signaling by activated HCA_2_ (gray arrow) remains poorly understood but is likely indirect (Offermanns and Schwaninger, 2015).

Of the currently approved therapies for MS, DMF (Tecfidera^®^) is one of the most promising. Its rapid approval followed two large-scale phase-III trials demonstrating the benefit of DMF treatment in relapsing MS patients (Fox et al., 2012; Gold et al., 2012). DMF is an analog of the electrophilic metabolite fumarate and is believed to operate through LDE-signaling-like mechanisms. However, DMF was approved with no clear MOA, and critical questions still abound as to the most relevant molecular target(s) and downstream players necessary and sufficient for therapeutic output. What is clear from studies in animal models and MS patients is that DMF functions through suppression of inflammation and complex modulation of the immune system (Yadav et al., 2019). However, DMF also has detrimental systemic side effects likely arising from off-target protein modifications. Thus, understanding the precise MOA is the key to the development of improved small-molecule therapeutics for MS. We here summarize select targets/pathways implicated in DMF’s MOA (Figure 2), with a view toward the role that electrophile regulation plays in therapeutic effects.

### Keap1/Nrf2/AR Signaling

As the master LDE sensor, Keap1 has long been suspected as a key DMF target. Nrf2-driven genes are upregulated (10–30% from baseline) in whole blood samples from MS patients at 12 weeks post-DMF treatment (Gopal et al., 2017). Similarly, cultured human, mouse, and rat astrocytes treated with monomethyl fumarate (MMF, to which DMF is rapidly converted by esterases in the body) or DMF upregulate Nrf2 protein levels and Nrf2-driven genes; knockdown of Nrf2 suppresses these effects (Linker et al., 2011). In mice subjected to attenuated EAE, Nrf2-knockout (KO) ablates the ~20% reduction in clinical score (CS) upon DMF treatment, but CS is already increased by ~40% in the non-DMF-treated Nrf2-KO animals relative to wild type (WT), complicating data interpretation.

Because these studies sought to elucidate the role of Keap1 modification/Nrf2 activation under conditions of bolus D(M)MF administration wherein multiple proteins are modified, questions about the validity of these results have been raised. A study using an EAE model designed to mimic the most inflammatory phase of the disease found that *both* WT and Nrf2-KO mice feature a 40% reduction in mean CS following DMF treatment, compared to that in the controls (Schulze-Topphoff et al., 2016). The authors also replicated the attenuated EAE model used previously and demonstrated Nrf2 independence of CS reduction by DMF in this model, consistent with their data in acute EAE mice. These results likely imply that less subjective (and/or complementary) parameters than/to CS should be used to derive important conclusions. Trying to build a complete understanding from these disparate data is further complicated by the inconsistent use of DMF and MMF, which feature distinct pharmacokinetic properties (Mrowietz et al., 2018).

Overall, although Nrf2 is clearly activated by DMF, this activation fails to fully account for the therapeutic effects. Ultimately, invoking Nrf2 activation (a cytoprotective and cell survival response) is somewhat contradictory to the clinically relevant effects of DMF, i.e., immune cell apoptosis (Treumer et al., 2003). The latest efforts have thus been devoted to identification of additional targets/pathways relevant for DMF’s MOA beyond Keap1/Nrf2. However, as summarized below, no pathways/players reported to date are able to fully account for DMF-induced selective immune cell depletion.

### PKCθ

Recent efforts have utilized ABPP to mine the proteome for novel targets of DMF in activated primary human and mouse T cells (Blewett et al., 2016). This experiment profiled ~2,400 cysteines (~1% of total protein cysteines in the human genome; Long and Aye, 2017; Hoch et al., 2018), from which 52 cysteines (from 49 proteins) with ABPP ratio (DMF/control) >4 were designated as hits. The authors chose to study the kinase PKCθ because of its role in the activation of T cells, which is inhibited by DMF. Two cysteines were modified upon DMF treatment: C322 (ABPP ratio 1.65) and either C14 or C17 (ABPP ratio 4.21). Because C14 and C17 are housed within the same tryptic peptide, the specific target cysteine was not identified. DMF treatment of activated PKCθ–KO T cells expressing PKCθ (WT or C14S/C17S) impairs the association of WT-PKCθ with CD28 (the event required for T-cell activation), but (C14S/C17S)-PKCθ is unresponsive. Critically, however, secretion of IL-2 is suppressed upon DMF treatment of *both* WT- and (C14S/C17S)-expressing cells, albeit to a lesser extent for the mutant (approximately 5-fold suppression vs. ~12-fold for WT). Therefore, PKCθ is insufficient to fully account for DMF-induced output. Because the study was performed in isolated T cells, the contribution of PKCθ to the MOA of DMF (a metabolically active drug) *in vivo* remains unsettled.

### GAPDH

Beyond studying potential targets profiled by proteomics strategies, known LDE sensors have also been investigated for relevance in DMF’s MOA. GAPDH, a key glycolytic enzyme, reacts with LDEs (e.g., HNE), leading to enzyme inhibition (Uchida and Stadtman, 1993). A study using LC/MS analysis of purified GAPDH incubated with fumarate identified C149 (catalytic Cys) and C244 modifications (rat protein residue numbering; Blatnik et al., 2008). Notably, prolonged (1 h) treatment of GAPDH (~25 μM) with high concentrations of fumarate (50 mM) is required to achieve 80% inhibition. The same cysteines are modified (by 10–15%) by endogenous fumarate in GAPDH enriched from rat muscles, raising the question of ligand occupancy on GAPDH–cysteine(s) available/required for therapeutic response following administration of fumarate-analog drugs.

A more recent study sought to link D(M)MF modification of GAPDH to immune modulation (Kornberg et al., 2018). Oral administration of D(M)MF to mice leads to monomethyl succination of the conserved catalytic-Cys in GAPDH (C150, mouse numbering); the corresponding cysteine (C152, human numbering) is modified in peripheral blood mononuclear cells (PMBCs) derived from MS patients. IL-1β production and translocation of NF-κB in peritoneal macrophages, and differentiation of T cells are affected by DMF. However, conclusive links between these effects and precise target engagement remain limited as the authors relied on another electrophilic GAPDH inhibitor, heptelidic acid (Figure 1E; Endo et al., 1985), as a proxy for GAPDH inhibition by D(M)MF. Heptelidic acid treatment reduces EAE CS by ~30%, on par with experiments using D(M)MF (Schulze-Topphoff et al., 2016). However, as covalent inhibitors often have vastly different pharmacokinetics and off-target spectra, despite their apparent chemical similarity and/or a common protein target, the use of a different electrophile precludes unambiguous assignment of GAPDH as a key player in the MOA of D(M)MF.

### HCA_2_

Hydroxycarboxylic acid receptor 2 (HCA_2_; also known as GPR109A) is a G protein-coupled membrane receptor. HCA_2_ activation downregulates lipolysis (Offermanns and Schwaninger, 2015) and inhibits inflammation through suppression of NFκB signaling (Zandi-Nejad et al., 2013). MMF is a known agonist of HCA_2_ with a similar EC_50_ of activation to that of nicotinic acid (9.4 and 2 μM, respectively), a well-characterized agonist (Tang et al., 2008). HCA_2_-KO EAE mice show no significant change in CS upon DMF treatment compared to ~60% reduction for WT animals (Chen et al., 2014). DMF reduces neutrophil infiltration into EAE lesions in WT mice by 30% but not in HCA_2_-KO mice. Demyelination is not reduced in HCA_2_-KO mice upon DMF treatment, although HCA_2_-KO alone, without DMF treatment, decreases demyelination by ~35% compared to the ~50% reduction measured in DMF-treated WT animals. Induction of some Nrf2-driven genes by DMF is impaired upon HCA_2_-KO, which, given the data discussed above on Nrf2 independence in DMF’s MOA (Schulze-Topphoff et al., 2016), may imply DMF-induced crosstalk between these pathways. It would be interesting to address whether membrane-targeted G-REX would detect HCA_2_ as a KPS of DMF and subsequently deploy T-REX to dissect potential crosstalk between modified HCA_2_ and Keap1/Nrf2/AR. Feasibility of such an analysis is supported by our recent data illuminating a nuanced LDE-modulated crosstalk among Keap1/Nrf2/AR, β-TrCP, and Wnt pathways, that is masked under bolus electrophile-dosing strategies (Long M. J. et al., 2017).

### p65 and IκBα

NFκB signaling is another well-appreciated LDE-modulated pathway. The NFκB transcription factor p65 is modified by alkyne-functionalized DMF (Kastrati et al., 2016). In MCF7 cells, DMF suppresses NFκB activity by 75%, and this effect is abrogated in cells expressing the sensing-defunct-mutant (C38S)-p65. Inhibition stems from a suppression of p65 nuclear translocation and DNA binding ability upon DMF modification. Similar results have been reported using U2OS cells (Gillard et al., 2015). IκBα, the negative regulator of NFκB and a known LDE sensor, may also be targeted by DMF, although direct modification has not yet been demonstrated (Dou et al., 2012). Overall, the relevance of NFκB signaling in DMF’s MOA has not been explicitly evaluated experimentally in MS patients or animal models, although the relative benefits, drawbacks, and challenges of modulation of this pathway in MS have been reviewed extensively (Leibowitz and Yan, 2016).

### RSK/MSK Kinases

DMF reportedly inhibits MSK and RSK kinases, downstream effectors in MAP-kinase cascades (Gesser et al., 2011). *In vitro*, DMF (but not MMF) inhibits RSK2 in a dose-dependent manner (Andersen et al., 2018). Co-crystallization of the C-terminal kinase domain of RSK2 with excess DMF revealed C599 and C436 modifications. *In vitro* and in cells, mutation of C599 to valine ablates DMF-induced RSK2 inhibition. The kinetics of DMF adduction of RSK2 appear to be quite slow (48-h treatment yielding ~50% inhibition). Typical second-order target-adduction rates for approved covalent drugs (*k*_inact_/*K*_i_~10^4^–10^6^ M^−1^ s^−1^; Schwartz et al., 2014; Parvez et al., 2018)] cast doubt on RSK2 as a physiologically relevant target. Since access to C599 is obscured upon kinase activation (based on the crystal structure), and as ERK signaling (upstream of RSK) is chronically upregulated in MS (Kotelnikova et al., 2019), the extent to which C599 is accessible for DMF target engagement remains unclear. These studies further highlight that data obtained using supraphysiological amounts of LDEs/drugs must be interpreted carefully: of the two cysteines modified under co-crystallization conditions, only one transpired to be (potentially) functionally relevant.

## LDE Signaling in Stress Defense, Aging, and MS Etiology

Age-related disorders share several interesting overlaps with autoimmune diseases such as MS, particularly in the CNS. The aging process is characterized by accumulation of molecular damage by reactive species, reminiscent of the damage that occurs in MS lesions (Ogrodnik et al., 2019). Not surprisingly, LDEs accumulate with age in the brain. For instance, protein adducts with the LDE malondialdehyde range from 1.5- to 2-fold higher in various regions of the brains of old-age individuals compared to middle-age individuals (Domínguez-González et al., 2018). Studying the mechanisms by which these age-dependent accumulations occur may offer insight into disease stage-dependent LDE accumulations that have been observed in MS lesions (Haider et al., 2011). Although LDEs that accumulate in aging and MS have long been regarded as toxic, damaging species, their roles as signaling mediators, particularly in cytoprotective and stress-defense pathways, are now well appreciated (Parvez et al., 2018).

Many outstanding questions remain as to how and why LDE signals maintain organismal homeostasis, and how such protective mechanisms relate to aging and MS pathophysiology. On the one hand, endogenous LDE accumulation is appreciated to be localized and generally context specific (Schopfer et al., 2011). Localized stress responses (Fivaz and Meyer, 2003) play critical roles for local governance of neuronal cell function and subcellular homeostasis, such as localized protein synthesis in extended axons and dendrites (Holt et al., 2019). Beyond proteome-level responsivity to localized production of stress signals, transcriptome-level operations such as mRNA trafficking in neurons are also acutely responsive to subcellular stress (Nussbacher et al., 2019). Given the heightened significance of LDE-related electrophilic drugs, the ability to precisely perturb and probe LDE-associated stress response mechanisms and players that support these subcellular activities will be a major step forward toward understanding how context-dependent deregulation occurs in aging (Castelli et al., 2019) and in MS. For instance, G-REX targeted to particular subcellular locales in neurons can profile first-responder proteins to localized LDE generation. Although the compatibility of REX technologies with complex vertebrate animals such as larval zebrafish is encouraging (Long M. J. C. et al., 2017), zebrafish MS models are still in their infancy (Burrows et al., 2019). Future tool development and effort should thus focus on multidiscplinary approaches such that LDE signaling can be studied in the most biomedically relevant context.

## Conclusions and Outlook

Functionally sufficient molecular targets and genetically necessary downstream players in DMF’s MOA altogether remain murky. General issues that persist in the LDE signaling field (Parvez et al., 2018) further cloud our understanding. Still, DMF is, to date, a promising small-molecule-based treatment for MS, and a second-generation DMF analog, diroximel fumarate (Vumerity^®^), has recently been approved by the FDA (US Food and Drug Administration, 2019; Figure 1E). As more electrophilic pharmaceuticals come onto the scene, demand is growing for better understanding of the functionally relevant targets of these reactive pleiotropic ligands and the mechanisms by which they exert their beneficial (or adverse) responses. The electrophile-signaling field is uniquely poised to offer critically needed insights into these novel pharmacophores, and the suite of tools now available will doubtless play a key role in these ongoing and future endeavors.

## Data Availability Statement

All datasets generated for this study are included in the article/supplementary material.

## Author Contributions

Both authors wrote and critically revised the manuscript.

## Conflict of Interest

US patent application has been filed for the G-REX method. The authors declare that the research was conducted in the absence of any commercial or financial relationships that could be construed as a potential conflict of interest.
